# Abstracts of Papers Presented at the Sixth Biennial Scientific Meeting of the Asia Pacific Paediatric Endocrine Society

**DOI:** 10.1155/2010/358358

**Published:** 2010-11-14

**Authors:** Paul Hofman

**Affiliations:** 1 Liggins Institute, New Zealand

## Publisher note

To access the full article, please see PDF.

